# The role of DSM-5 borderline personality symptomatology and traits in the link between childhood trauma and suicidal risk in psychiatric patients

**DOI:** 10.1186/s40479-017-0063-7

**Published:** 2017-06-18

**Authors:** Bo Bach, Rita Fjeldsted

**Affiliations:** 1 Center of Excellence on Personality Disorder, Psychiatric Research Unit, Slagelse Psychiatric Hospital, Fælledvej 6, 4200 Slagelse, Denmark; 2 Psychiatric Clinic, Slagelse Psychiatric Hospital, Slagelse, Denmark

**Keywords:** Borderline personality disorder, Childhood trauma, Child abuse, Child maltreatment, Suicidal risk, Suicidality, DSM-5 Section III personality traits, Personality inventory for DSM-5 (PID-5), Dissociation, Depressivity

## Abstract

**Background:**

Childhood traumas appear to be linked to suicidal behavior. However, the factors that mediate between these two phenomena are not sufficiently understood. Recent findings suggest that borderline personality disorder (BPD) may explain some of the association.

**Method:**

The present study investigated the potential mediating role of BPD symptomatology and traits between reported childhood trauma and suicidal risk in adult psychiatric outpatients (*N* = 124). BPD symptomatology was measured with DSM-5 Section II criterion-counts (SCID-II; Structured Clinical Interview for DSM-IV Axis II), whereas BPD traits were measured with specified DSM-5 Section III traits (PID-5; Personality Inventory for DSM-5). Childhood traumas were self-reported (CTQ; Childhood Trauma Questionnaire), whereas level of suicidal risk was measured with a structured interview (MINI Suicidality Module; Mini International Neuropsychiatric Interview). Mediation effects were tested by bias-corrected (10.000 boot-strapped samples) confidence intervals.

**Results:**

BPD features account for a considerable part of the cross-sectional association between childhood trauma and level of suicidal risk, even when controlling for the influence of gender, age, and educational level. This finding remained stable when testing the model without the suicidality-related BPD criterion and PID-5 items. DSM-5 Section II BPD criterion-counts explained 67% of the total effect, whereas DSM-5 Section III BPD traits accounted for 82% of the total effect. The specific DSM-5 Section III trait facets of “Depressivity” (52%) and “Perceptual Dysregulation” (37%) accounted for most of this effect.

**Conclusions:**

The findings provide preliminary support for the proposed mediation model indicating that BPD features may help explain relations between childhood trauma and elevated suicidal risk in adult life, in particular for DSM-5 Section III personality traits of depressivity (e.g., pessimism, guilt, and shame) and perceptual dysregulation (e.g., dissociation). To reduce the suicidal risk among those with a history of childhood trauma, BPD features (including “Depressivity” and “Perceptual Dysregulation”) might be an important target of assessment, risk management, and treatment. However, other factors are likely to be involved, and a longitudinal and more large-scale design is warranted for a conclusive test of mediation.

**Electronic supplementary material:**

The online version of this article (doi:10.1186/s40479-017-0063-7) contains supplementary material, which is available to authorized users.

## Background

Suicide is one of the main causes of death around the world [1]. The risk of suicide is particularly predominant among patients in mental health care or psychiatric settings [2], and is often related to the presence of Borderline Personality Disorder [BPD] [3, 4]. Moreover, a vast amount of research suggests that a comprehensive understanding of suicidal behavior must take childhood adversities into account as there is strong support for a significant relationship between childhood traumas and suicidal risk in adulthood, even when controlling for the effects of other mental disorders [5, 6]. Essentially, numerous studies support that childhood traumas contribute to the development of personality pathology [7–9], which in turn has been found to predict the onset, course, and recurrence of suicidal behavior [6, 10, 11]. This suggests that childhood traumas affect the risk for suicide by being a determinant of enduring liabilities or maladaptive personality features such as BPD [10]. In support of this, it has been demonstrated that PD criterion counts (including BPD) mediate the cross-sectional relationship between childhood adversity and depressive disorders including feelings of hopelessness and suicidal ideations [12, 13]. In one cross-sectional study among college students, it was found that BPD symptomatology is a specific mediator of the link between child maltreatment and adult suicide potential [14]. In another study, a structural equation model analysis confirmed that BPD features have a mediating role between adverse life-historical events and symptomatic disorders among suicidal psychiatric patients [10]. Overall, a number of studies indicate that personality pathology in general play a mediating role in the relationship between childhood traumas and symptom distress (including suicidal aspects). This particularly applies to the trait of neuroticism, which is also considered a core trait in BPD [15]. Importantly, these mediating relationships have not only been supported in cross-sectional research [16] but also in longitudinal studies [17], and even when the supposed genetic effect on both personality and psychopathology is accounted for [6, 18]. For example, a prospective twin-study by Kendler & Gardner [19] showed that personality traits mediate the link between risk factors (genes and childhood environmental stressors) and symptoms of depression. In the current study, we specifically expected BPD to meditate this relationship as it particularly has been found to increase the risk of suicidality [20] while also being strongly related to childhood adversities [21]. Additionally, research suggests that childhood abuse is a core risk factor for suicidal behavior in patients with BPD [22]. To date, only a couple of studies have investigated the specific role of BPD in this proposed mediation model. Additionally, to our knowledge, no previous study has investigated which specific BPD features (i.e., traits) that may exert the mediating effect. This seems important to delineate because BPD involves a highly heterogeneous constellation of pathological features. Finally, no previous study has examined the mediating role of the DSM-5 Section III trait operationalization of BPD features in the aforementioned relationship. The current study sought to fill out these gaps.

The aim of the present study was to examine the potential mediating role of BPD along with specific BPD features in the relationship between childhood trauma and suicidal risk in adult psychiatric outpatients. This was approached by employing interview-rated DSM-5 Section II BPD criteria as well as self-reported DSM-5 Section III BPD traits, simultaneously. Thus, we proposed preliminary meditational models in which BPD and specific BPD features mediate the relationship between childhood traumas and suicidal risk in adulthood. Accordingly, the formal objective of the current study was to test these models using self-reported childhood traumas, interview-rated level of suicidal risk, interview-rated DSM-5 Section II BPD criteria, and self-reported DSM-5 Section III BPD traits.

## Method

### Participants and procedure

A sample of nonpsychotic outpatients (*N* = 124) were consecutively recruited (by means of a naturalistic design) from a psychiatric hospital unit in Denmark specialized in the assessment and treatment of personality disorders. Sociodemographic characteristics and levels of suicidality are provided in Table 1. All patients were administered diagnostic interviews for common mental disorders, personality disorders (including BPD criteria), and suicidal risk as well as self-report inventories measuring childhood trauma and DSM-5 Section III pathological personality traits. The diagnostic interviews were performed by a trained clinical psychologist under the supervision of a psychiatrist. Patients suspected of having a current organic disorder, substance-induced condition, psychotic disorder, severe depression, autism, or manic episode were not included. As presented in Table 2, the sample was predominantly characterized by BPD. All self-reported data were collected via secure online self-report software, that did not allow the respondents to skip any items, which prevented missing data. As a part of their treatment program all patients received individual feedback on their SCID-II and PID-5 profiles. All participants gave consent to have their data used for research.

**Table 1:** Sociodemographic characteristics of the 124 patients

Gender; *n* (*%*)	
Female	97 (78.2%)
Male	27 (21.8%)
Age; years	
Mean (*SD*)	28.9 (8.45)
Range	18 – 56
Relationship status; *n* (%)	
In a relationship	72 (58.1%)
Single	52 (41.9%)
Occupational status; *n* (%)	
Employed^a^	24 (19.4%)
Unemployed^b^	100 (80.6%)
Educational level; *n* (%)	
Above bachelor level	6 (4.8%)
At bachelor level	9 (7.3%)
Below bachelor level	47 (27.9%)
No education	62 (50.0%)
Psychiatric Services History; *n* (%)	
Acute/emergency admission	48 (38.7%)
Inpatient admission	46 (37.1%)
Outpatient admission	55 (44.4%)
Lifetime Suicidality; *n* (%)	
Never had any suicidal ideations	13 (10.5%)
Have contemplated suicide	36 (29.0%)
Have planned suicide	12 (9.7%)
Have attempted suicide	63 (50.8%)

^a^Student, employee, or self-employed; ^b^includes long-term sick leave and disability pension

**Table 2:** Characteristics of DSM-5 section II personality disorders and mental disorders

	Personality disorder	*N* (%)	Mental disorders	*N* (%)
A	Paranoid	61 (49.2%)	Major Depressive Disorder	36 (29.0%)
	Schizotypal	10 (8.1%)	Dysthymia	38 (30.6%)
	Schizoid	5 (4.0%)	Social Phobia	58 (46.8%)
			Post-Traumatic Stress Disorder	39 (31.5%)
B	Borderline	89 (71.8%)	Panic Disorder	52 (41.9%)
	Narcissistic	3 (2.4%)	Agoraphobia	66 (53.2%)
	Histrionic	0 (0.0%)	Obsessive Compulsive Disorder	42 (33.9%)
	Antisocial	22 (17.7%)	Anorexia Nervosa	5 (4.0%)
C			Bulimia Nervosa	26 (21.0%)
	Avoidant	65 (52.4%)	Generalized Anxiety Disorder	25 (20.2%)
	Dependent	18 (14.5%)	Substance Use Disorder	13 (10.5%)
	Obsessive-Compulsive	40 (32.3%)	Alcohol Use Disorder	10 (8.1%)
			Lifetime Psychotic Episode	14 (11.3%)
	Not Otherwise Specified	6 (4.8%)	No criteria met	1 (0.8%)
	No criteria met	7 (5.6%)		

*N* = 124

## Measures

### DSM-5 Section II BPD criteria

The DSM-5 Section II criteria for BPD were systematically assessed using the Structured Clinical Interview for DSM–IV Axis II Personality Disorders [SCID-II] [23]. BPD features were measured dimensionally by adding the number of fulfilled criteria for each category (criterion count). All SCID-II interviews were performed and recorded independently of the computerized administration and scoring of the self-reported childhood traumas and pathological personality traits. Thirteen randomly selected SCID-II interviews were inter-rated by a blinded psychologist, and optimal interrater reliability was identified on the basis of criterion-counts, with a two-way random intra-class correlation coefficient for single rater of .977 (confidence interval = .927-.993; *p* > .001). See Additional file 1: Table S1 and Additional file 2: Table S2 for base rates and distribution of BPD diagnostic criteria.

### DSM-5 Section III BPD traits

The DSM–5 Section III traits for BPD were assessed using the Personality Inventory for DSM-5 [PID-5] [24, 25]. The PID-5 is a 220-item self-report inventory measuring the Criterion B (i.e., 25 trait facets and five higher-order trait domains) of the alternative DSM–5 model for PDs. Patients were required to rate each PID-5 item on a 4-point likert-scale from 0 (Very False or Often False) to 3 (Very True or Often True). We used the Danish version of the PID-5, which has demonstrated acceptable psychometric properties [26, 27] and continuity with categorical PDs [28], including specific ability to differentiate BPD from other PDs [29, 30]. In the current study we exclusively employed 9 PID-5 trait facets that were designated to describe features of BPD. First, we included the 7 trait facets that are specified for BPD in the official DSM-5 Section III trait-to-disorder cross-walk (i.e., Emotional Lability, Anxiousness, Separation Insecurity, Depressivity, Impulsivity, Risk Taking, and Hostility [31]). Additionally, we included Suspiciousness and Cognitive & Perceptual Dysregulation reflecting the ninth Section II diagnostic criterion of BPD comprising stress-induced paranoid ideations and/or dissociative experiences, which has been supported by previous findings [29, 30, 32–34]^1^. In the present study, alpha coefficients for the nine facets ranged from .78 (Emotional Lability) to .92 (Depressivity) with a mean alpha of .87.

### Level of suicidal risk

Suicidal risk was assessed using the “Suicidality” module of Mini International Neuropsychiatric Interview 6.0 (MINI; Sheehan et al. [35]). In this structured interview module, the patients are probed for various aspects of suicidality and suicide risk factors within the last month (e.g., suicidal thoughts or plans, deliberate self-harm with or without expectation to die, and history of suicidal attempts). Subsequently, the obtained information are added up and scored as “Low”, “Moderate”, or “High” suicide risk. The suicidality module of the MINI diagnostic interview has demonstrated sound psychometric and predictive features in relation to suicidality and risk assessment [35, 36].

### Childhood trauma

Childhood traumas were measured with the Childhood Trauma Questionnaire (CTQ; Bernstein & Fink, [37]). The CTQ is a 25-item self-report inventory (excluding 3 validity items) containing retrospective questions concerning childhood abuse and neglect. On a 5-point Likert-type scale (“Never true” to “Very often true”) the respondent indicates how well each item reflects what they have experienced during their childhood. In the current study we computed a total score by averaging all item responses. A number of international studies suggests that the reliability and validity of the CTQ is excellent [38]. In the present study, the total CTQ scale score had an alpha coefficient of .93, and a mean corrected item-total coefficient of .57 with all coefficients above .40 (except “item 9” [.37] describing severe physical violence).

## Results

### Bivariate associations

Table 3 displays bivariate associations among all study variables along with descriptive statistics. All the variables demonstrated significant intercorrelations, except the DSM-5 Section III BPD trait of Risk taking. Accordingly, the CTQ score was significantly related to BPD criterion-count score, which was significantly related to level of suicidal risk. Likewise, childhood trauma was significantly related to the PID-5 facets of Anxiousness, Depressivity, Hostility, Impulsivity, Suspiciousness, and Perceptual Dysregulation, which were also significantly related to level of suicidal risk. For childhood traumas the strongest associations applied to the SCID-II BPD criterion count score (.42) along with PID-5 facets of Suspiciousness (.39), Perceptual Dysregulation (.37), and Depressivity (.30). For suicidal risk, the strongest associations applied to PID-5 facets of Depressivity (.53), Perceptual Dysregulation (.39), and Separation Insecurity (.38) as well as SCID-II BPD criterion count score (.37).

**Table 3:** Bivariate associations among study variables

		1.	2.	3.	4.	5.	6.	7.	8.	9.	10.	11.	M (SD)
1.	MINI Level of Suicidal Risk												2.67 (1.03)
2.	CTQ Childhood Trauma	.22											2.17 (0.73)
3.	SCID-II BPD criterion count	.37	.42										5.65 (2.41)
4.	PID-5 Emotional Lability	.30	.06	.49									1.80 (0.65)
5.	PID-5 Separation Insecurity	.38	.12	.44	.45								1.34 (0.90)
6.	PID-5 Anxiousness	.31	.19	.40	.53	.49							1.91 (0.67)
7.	PID-5 Depressivity	.53	.30	.45	.43	.45	.58						1.53 (0.70)
8.	PID-5 Hostility	.23	.20	.53	.33	.41	.34	.25					1.32 (0.67)
9.	PID-5 Impulsivity	.24	.20	.51	.30	.27	.09	.21	.45				1.10 (0.74)
10.	PID-5 Risk Taking	.09	.14	.21	-.15	-.05	-.26	-.05	.25	.40			1.10 (0.64)
11.	PID-5 Suspiciousness^a^	.34	.39	.65	.44	.48	.52	.56	.58	.36	.11		1.49 (0.80)
12.	PID-5 Perceptual Dysregulation^a^	.39	.37	.39	.25	.20	.38	.41	.34	.32	.28	.46	0.86 (0.53)

*N* = 124; *MINI* Mini International Neuropsychiatric Interview 6.0 – Suicidality Module, *CTQ* Childhood Trauma Questionnaire (total score), *SCID-II* Structured Clinical Interview for DSM-IV Axis II Borderline Personality Disorder, *PID-5* Personality Inventory for DSM-5. Correlations from .19 are significant at the 0.05 level and correlations from .30 are significant at the 0.001 level. ^a^PID-5 facets that were added to the operationalization of BPD based on empirical findings [29, 30, 32, 33]

### Mediational analysis

We employed mediation analysis to explore the mediating effects of DSM-5 Section II BPD criterion-count and DSM-5 Section III BPD traits, separately, in the relationship between childhood trauma and level of suicidal risk. In mediation analysis different effects are considered: The total effect of an independent variable (IV) on a dependent variable (DV) is composed of the direct effect of the IV on the DV and the indirect effect through a proposed mediator variable. First, we examined the direct and indirect effect of childhood trauma on level of suicidal risk through DSM-5 Section II BPD criterion count. Next, we examined the direct and indirect effect of childhood trauma on level of suicidal risk through the nine designated DSM-5 Section III BPD traits, simultaneously, while controlling for the potential mediating effects of the other traits. Based on recommendations by Hayes [39] and Mackinnon et al. [40], a bootstrapping sampling procedure was applied for assessing effects. Importantly, bootstrapping is a nonparametric approach that can provide more accurate inferences when the data are not well behaved, as in the current study, in terms of distributional assumptions (e.g., non-normality) and when the sample size is small [41]. The reported coefficients and effects were considered significant if zero was not included in the 95% bias-corrected confidence interval. All effects were adjusted for the influence of gender, age, and educational level (see Table 1). Multicollinearity of independent variables was unlikely to be an issue (all VIF < 2.5; tolerances > .40). In the current study we used PROCESS version 1.12.1 for SPSS provided by Hayes [39].

**Fig. 1:**
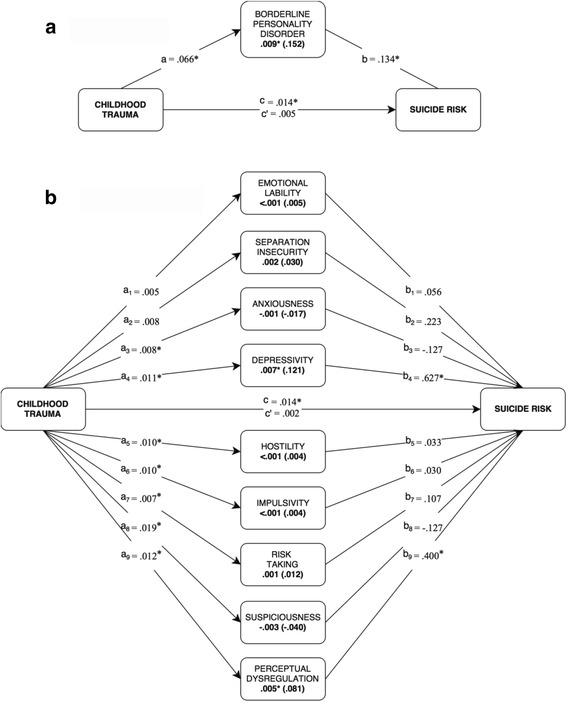
Mediation analyses of BPD features between Childhood Trauma and Level of Suicidal Risk. *Note. N* = 124 patients. Bolded values are indirect/mediating effects, with standardized effects in parentheses. The a-, b- and c-paths represent regression coefficients. *c* = total effect, *c’* = direct effect (when *a* and *b* are accounted for). All coefficients marked with an asterisk (*) are significant in terms of 95% bias-corrected confidence intervals that do not contain zero (10,000 bootstrapped samples). In mediation model B, all mediator variables (i.e., PID-5 scales) were analyzed simultaneously while controlling for the effect of the other scales. Estimations were statistically adjusted for age, gender, and educational level. **a** DSM-5 Section II. **b** DSM-5 Section-III Traits

The mediational model for DSM-5 Section II BPD criterion-count is displayed in Fig. 1a. Both the a-path and the b-path were significant, while the c-path (total effect) did not remain significant when the effect of BPD was taken into account (c'-path; direct effect). As presented in Additional file 3: Table S3, the BPD criterion-count score explained 67% of the total effect of childhood trauma on suicide risk. We further tested the specificity of BPD as mediator by redoing the analyses using each of the remaining 9 PDs as mediating variables, individually, which only yielded significant but inferior effects for Dependent PD (.002), Paranoid PD (.002), and Schizotypal PD (.003). Subsequently, when performing a mediation analysis with Borderline, Dependent, Paranoid, and Schizotypal PDs in one simultaneous model, only the effects of Borderline (.010) and Dependent (.002) remained significant. This suggests that BPD is the core mediator of the relationship in question.

The mediational model for DSM-5 Section III BPD traits is displayed in Fig. 1b. The trait facets of Depressivity and Suspiciousness showed significant a-paths and b-paths, and the c-path (direct effect) did not remain significant when controlling for the effect of BPD trait facets. As presented in Additional file 3: Table S3, the total DSM-5 Section III BPD composite trait score accounted for 82% of the total effect of childhood trauma on suicidal risk. Specifically, the trait facet of Depressivity explained 52% whereas Perceptual Dysregulation explained 37% of this effect. As the PID-5 facets of Suspiciousness and Perceptual Dysregulation are not included in the official definition of the DSM-5 Section III BPD type, we tentatively retested the model without these two facets. Consequently, the total indirect effect remained significant and relatively stable (decreased from .012 to .010), and the direct effect remained non-significant. Apart from the facet of Depressivity no other facets emerged as significant mediators in this model.

As both DSM-5 Section II (BPD Criterion 5; "recurrent suicidal behavior, gestures, or threats, or self-mutilating behavior") and Section III (PID-5 Depressivity facet; items 81, 119, and 178) explicitly refer to potential suicidality, it may not seem surprising that these features are associated with suicidal risk. Therefore, to test for possible circularity we also performed the analyses without this suicidality-related content. First, we computed a BPD criterion count without criterion number 5, and included this modified score in the mediation model. Notably, the mediating effect of BPD remained significant and was only reduced from .009 to .007. Second, when excluding the PID-5 Depressivity items that explicitly refer to suicidality (items 81, 119, and 178), the mediating effect of Depressivity remained significant but decreased from .007 to .005, whereas the mediating effect of perceptual dysregulation increased slightly from .0047 to .0053; no other facets emerged as significant mediators in this model. In this modified model, the total indirect effect of all 9 PID-5 facets decreased slightly from .0118 to. 0113 and remained significant, and the direct effect remained non-significant.

**Fig. 2:**
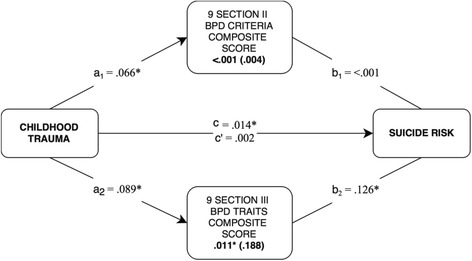
Parallel mediation analysis comparing the indirect effect of DSM-5 Section II versus Section III BPD composite scores. *Note. N* = 124 patients. Bolded values are indirect/mediating effects with standardized effects in parentheses. The a-, b- and c-paths represent regression coefficients. Coefficients marked with an asterisk (*) are significant in terms of 95% bias-corrected confidence intervals that do not contain zero (10,000 bootstrapped samples). *c* = total effect, *c’* = direct effect (when *a* and *b* are accounted for). The two mediator variables (i.e., BPD scores) were analyzed simultaneously while controlling for the effect of one another. The contrast between the two BPD measures in terms of mediating effect was -.011*. Estimations were statistically adjusted for age, gender, and educational level

As an additional post hoc analysis, we also examined the mediational effect of DSM-5 Section II BPD criterion count versus DSM-5 Section III BPD composite score in one joint parallel mediation model (see Fig. 2) while controlling for the effect of one another. In this model, only DSM-5 Section III traits showed significant a-paths and b-paths, whereas the DSM-5 Section II traits only showed significant a-paths (association between childhood trauma and BPD). The contrast between the two mediational paths (.011) was statistically significant suggesting that the explanatory power of DSM-5 Section III traits is superior to DSM-5 Section II criterion-counts in terms of mediational effect.

## Discussion

In the present study we set out to test two proposed models in which DSM-5 Section II BPD criterion-count and DSM-5 Section III BPD traits mediate the effect of childhood traumas on suicidal risk in adult psychiatric patients. Overall, we found that childhood traumas were associated with the rated level of suicidal risk in terms of a small-moderate effect size. The indirect effect of DSM-5 Section II BPD criterion-counts accounted for 67% of the total effect of childhood traumas on suicidal risk, whereas the indirect effect of the DSM-5 Section III BPD composite score accounted for 82% of the total effect. Specifically, the DSM-5 Section III trait facets of Depressivity (52%) and Perceptual Dysregulation (37%) accounted for this effect. Importantly, these effects were also confirmed when suicidality-related content was excluded from the mediator variables. Finally, by comparing the mediating effect of Section II versus Section III BPD features in one parallel mediation model, we found that the Section III BPD trait model had substantially more explanatory power relative to the Section II BPD categorical model. This indicates that the trait operationalization of BPD may be more suitable for capturing features that lead to suicidality in comparison to the established categorical BPD model. Although, findings are based on statistical inferences from a cross-sectional sample, they underscore the significant role of BPD features as a potential mediator between childhood adversities and suicidal risk in adulthood. Accordingly, the proposed mediational models may be a reasonable basis for more comprehensive investigation in future studies. Moreover, as will be discussed in the following, the findings provide preliminary support for the proposed mediation model indicating that BPD features may help explain relations between childhood trauma and elevated suicidal risk in adult life, in particular for DSM-5 Section III traits of depressivity (e.g., pessimism, guilt, and shame) and perceptual dysregulation (e.g., dissociation).

Despite the fact that emotional dysregulation or emotional lability are viewed as a core feature of BPD [29, 30, 42], the current study only highlighted Depressivity and Perceptual Dysregulation as potential mediators of the link between childhood trauma and suicidal risk in adulthood. However, it may be argued that Depressivity and in particular Perceptual Dysregulation (i.e., dissociation proneness) comprise two manifestations of emotional dysregulation in terms of severe demoralization and stress-induced dissociation [43]. Accordingly, research has demonstrated that BPD patients exposed to stress were more prone to and showed higher levels of dissociation in comparison to patients with other mental disorders [44]. In other words, the level of suicidal risk in BPD may reflect BPD severity as indicated by elevated levels of Perceptual Dysregulation (i.e., dissociation or psychotic-like experiences) and Depressivity (i.e., self-loath or demoralization).

Our identification of Depressivity (including subfacets of guilt, low self-worth, shame, and pessimism) as mediator between childhood trauma and suicidal risk is largely consistent with previous studies investigating the role of depressive traits. For example, Whiffen and colleagues [45] found that personality features of self-criticism mediated the relationship between lack of parental care and symptoms of depression (including suicidality). Likewise, Swannell and colleagues [46] identified self-blame as mediator between child maltreatment and non-suicidal self-injury. It is hypothesized that child maltreatment may engender self-loath and shame, promoting self-blaming and pessimistic attributional style possibly due to internalizing parental (or other authorities inflicting maltreatment) negative beliefs about them [47]. The guilt, shame or pessimistic attributional style comprising the DSM-5 Section III trait of Depressivity may well increase vulnerability to using suicide as a management technique, such that suicidal behavior or attempt becomes a strategy to ease emotional pain and distress resulting from guilt, shame and pessimism.

Our identification of Perceptual Dysregulation (including features of dissociation-proneness) as mediator between childhood traumas and suicidal risk is consistent with previous studies investigating the role of dissociation. First, previous research indicates that early traumas provoke a structural dissociation of the premorbid personality [48], and that dissociative symptoms are positively related to current stressors [44]. Second, the finding also aligns with theory suggesting that childhood traumas disturb the normal development of cognitive and affective processing, integration of thinking and feeling, and capacity to understand and express emotional states, which give rise to problems such as dissociation [49]. It has further been suggested that childhood trauma disrupts normative progression toward use of language to share emotional experiences, requiring children to process trauma on a nonverbal level [50]. Finally, previous research has shown that features of dissociation mediate the effect of child sexual abuse on later non-suicidal self-injury [46]. Consequently, suicidal behavior may develop as a compensatory strategy to disrupt a sense of psychological numbing and/or to avoid and manage perceived uncontrollable emotions [49]. This is also in line with literature suggesting that clinician awareness of the frequency and severity of dissociated states in BPD is essential to safety planning in relation to suicidal risk management [43]. Thus, as a potential suicidal risk factor, Perceptual Dysregulation may explicitly reflect intrusive traumas that are linked to dissociation or psychotic-like experiences in the BPD patient.

### Strengths, limitations, and future directions

The main strength of the present study is the appropriate inclusion of psychiatric patients with BPD features, the utilization of DSM-5 Section II and III, and the employment of both self-report and interview-rated measures. This is both a significant and novel addition to understanding the potential role of BPD in relation to suicidal risk. However, certain limitations and recommendations for future research should be emphasized.

First, the use of mediation analysis on cross-sectional data has been questioned as one can find evidence for indirect effects in cross-sectional data, even when the true indirect effect in longitudinal data is zero, and vice versa [51]. However, in the present study mediation analysis was employed in accordance with recommendations by Hayes [39] and MacKinnon [40] as an initial attempt to test a proposed model. Moreover, our objective was also to compare the mediational effect exerted by DSM-5 Section II and II BPD features, respectively. Thus, the findings provided preliminary support for the model, but longitudinal research is necessary for a conclusive test of mediation. A more definitive test of our mediational model would require that childhood traumas be recorded during childhood and early adolescence, that BPD pathology be assessed during mid-adolescence, and that level of suicidal risk be assessed during adulthood. However, taken together with the many studies indicating that childhood traumas are associated with BPD, and that BPD predicts elevated suicidal risk, our findings suggest that future research may be likely to confirm that BPD, in particular features of Depressivity (i.e., shame, guilt, pessimism, and self-loathing) and Perceptual Dysregulation (i.e., dissociation) mediate a substantial part of the effect of childhood traumas on suicidal risk in adulthood.

Second, because childhood traumas were measured with a questionnaire, we were unable to differentiate between the effects of actual and perceived childhood events. It is possible that the results would have been different if reports from multiple informants (e.g., parents, siblings) had been available. Moreover, the retrospectively recollected reporting may have been influenced by state-dependent memory and recall bias reflecting personality and current mental condition. However, in a comparative study Scott et al. [52] found no significant difference between prospective records and retrospective self-reports of childhood maltreatment. Additionally, in the current study we did not assess inter-rater reliability of the MINI Suicidal Risk measure.

## Conclusions

The current study provided preliminary cross-sectional support for a mediational model in which DSM-5 BPD diagnostic features (interview-rated Section II BPD criteria and self-reported Section III BPD traits) potentially explain the link between childhood trauma and suicidal risk in adult life. Notably, the Section III BPD trait model outperformed the Section II BPD categorical model in terms of mediational effect. To reduce the suicidal risk among those with a history of childhood trauma, BPD-related problems (including “Depressivity” and “Perceptual Dysregulation”) might be important targets of risk assessment, psychoeducation, and treatment. However, other factors are likely to be involved, and a longitudinal and more large-scale design is necessary for a more conclusive test of mediation.

## Additional files

Additional file 1: Frequency distribution of 9 DSM-5 Section II BPD criteria. (PDF 409 kb)

Additional file 2: Base rates of 9 DSM-5 Section II BPD criteria. (PDF 481 kb)

Additional file 3: Mediation analyses of BPD features between Childhood Trauma and Level of Suicidal Risk. (PDF 304 kb)

## Abbreviations

BPD: Borderline personality disorder
DSM: Diagnostic and statistical manual of mental disorders
MINI: Mini international neuropsychiatric interview
PID-5: Personality inventory for DSM-5
SCID-II: Structured clinical interview for DSM-IV Axis II

## Footnotes

1 The rationale for including suspiciousness and perceptual dysregulation in the conceptualization of BPD is elucidated and empirically indicated elsewhere see [30, 32, 34].
