# Supplementary material for: The role of DSM-5 borderline personality symptomatology and traits in the link between childhood trauma and suicidal risk in psychiatric patients
Source: Borderline Personal Disord Emot Dysregul. 2017 Jun 18;4:12. doi: 10.1186/s40479-017-0063-7 (PMC5474295; doi:10.1186/s40479-017-0063-7)
Supplement: Supplementary file 1 — Frequency distribution of 9 DSM-5 Section II BPD criteria. (PDF 409 kb) [file 40479_2017_63_MOESM1_ESM.pdf]

Table S1: *Frequency distribution of 9 DSM-5 Section II BPD criteria*

**Number of fulfilled criteria**	**Frequency**	**Percent**	**Cumulative Percent**
0	5	4.0%	4.0
1	2	1.6%	5.6
2	8	6.5%	12.1
3	11	8.9%	21.0
4	8	6.5%	27.4
5	22	17.7%	45.2
6	15	12.1%	57.3
7	18	14.5%	71.8
8	23	18.5%	90.3
9	12	9.7%	100.0
Total	124	100%	-

Note. The median number of fulfilled BPD criteria was 6.0 (M = 5.65; SD = 2.41).
