# Supplementary material for: The role of DSM-5 borderline personality symptomatology and traits in the link between childhood trauma and suicidal risk in psychiatric patients
Source: Borderline Personal Disord Emot Dysregul. 2017 Jun 18;4:12. doi: 10.1186/s40479-017-0063-7 (PMC5474295; doi:10.1186/s40479-017-0063-7)
Supplement: Supplementary file 2 — Base rates of 9 DSM-5 Section II BPD criteria. (PDF 481 kb) [file 40479_2017_63_MOESM2_ESM.pdf]

Table S2: *Base rates of 9 DSM-5 Section II BPD criteria*

**DSM-5 Section II BPD Criteria**	***N***	**%**
1. Avoid Abandonment	76	61.3%
2. Unstable Relations	76	61.3%
3. Identity Disturbance	84	67.7%
4. Impulsivity	55	44.4%
5. Self-Destructiveness	90	72.6%
6. Affective Lability	98	79.0%
7. Feelings of Emptiness	83	66.9%
8. Inappropriate Anger	69	55.6%
9. Paranoia or Dissociation	71	57.3%
