# Supplementary material for: The role of DSM-5 borderline personality symptomatology and traits in the link between childhood trauma and suicidal risk in psychiatric patients
Source: Borderline Personal Disord Emot Dysregul. 2017 Jun 18;4:12. doi: 10.1186/s40479-017-0063-7 (PMC5474295; doi:10.1186/s40479-017-0063-7)
Supplement: Supplementary file 3 — Mediation analyses of BPD features between Childhood Trauma and Level of Suicidal Risk. (PDF 304 kb) [file 40479_2017_63_MOESM3_ESM.pdf]

Table S3. Mediation analyses of BPD features between Childhood Trauma and Level of Suicidal Risk

	Indirect Effect	BC **95**% CI		% of total indirect effect	% of total effect
		Lower	Upper		
**Section II BPD criterion count**	.009* (.152)	.062	.267	--	67%
**Section III BPD traits total**	.011* (.200)	.003	.020	--	82%
Emotional Lability	<.001 (.005)	-.017	.048	ns	--
Separation Insecurity	.002 (.030)	-.002	.110	ns	--
Anxiousness	-.001 (-.017)	-.093	.028	ns	--
Depressivity	.007* (.121)	.042	.243	60%	52%
Hostility	<.001 (.004)	-.058	.078	ns	--
Impulsivity	<.001 (.004)	-.048	.063	ns	--
Risk Taking	.001 (.012)	-.016	.073	ns	--
Suspiciousness	-.003 (-.040)	-.156	.065	ns	--
Perceptual Dysregulation	.005* (.081)	.011	.205	40%	37%

Note. Unstandardized effect sizes are reported, whereas standardized effects are in parentheses. Coefficients marked with an asterisk (*) are significant in terms of 95% bias-corrected confidence intervals that do not contain zero (10,000 bootstrapped samples). In the mediation model for Section III BPD traits, all mediator variables (i.e., PID-5 scales) were analyzed simultaneously while controlling for the effect of the other scales. Estimations were statistically adjusted for age, gender, and educational level.
